# “*As protective gear began to run low, guidance on protection became looser” -* Healthcare workers' perspectives on infection prevention and control during the COVID-19 pandemic

**DOI:** 10.3389/fpubh.2022.982738

**Published:** 2022-11-10

**Authors:** Ida Aulanko, Petra Nikuri, Lotta Oksanen, Sampo Oksanen, Laura Lahdentausta, Milla Pietiäinen, Susanna Paju, Anne Kivimäki, Pirkko Pussinen, Ahmed Geneid, Enni Sanmark

**Affiliations:** ^1^COVID19VATEHY Research Group, Head and Neck Center, Helsinki University Hospital, University of Helsinki, Helsinki, Finland; ^2^Doctoral Programme in Clinical Research, University of Helsinki, Helsinki, Finland; ^3^Joint Municipal Authority for Social and Healthcare in Central Uusimaa (Keusote), Hyvinkää, Finland; ^4^Department of Pediatrics, Helsinki University Hospital, University of Helsinki, Helsinki, Finland; ^5^Department of Health Sciences and Sport, University of Stirling, Stirling, United Kingdom; ^6^Department of Otorhinolaryngology and Phoniatrics, Head and Neck Surgery, Helsinki University Hospital, University of Helsinki, Helsinki, Finland; ^7^School of Business, Aalto University, Helsinki, Finland; ^8^Department of Oral and Maxillofacial Diseases, Helsinki University Hospital, University of Helsinki, Helsinki, Finland

**Keywords:** personal protective equipment, health personnel, surveys and questionnaires, communication, experiences

## Abstract

**Objectives:**

The COVID-19 pandemic has posed several risk factors to healthcare workers' (HCWs') emotional distress. The purpose of the study was to enhance understanding of the experiences and feelings of HCWs during the COVID-19 pandemic, with specific reference to infection prevention and control (IPC) practices and guidance, focusing on the quality and availability of personal protective equipment (PPE), guidelines, and management. With a qualitative approach, we aimed to enable a wider narrative; to gain a more detailed understanding related to PPE use and identify experiences that can be overlooked in forced-choice questionnaires.

**Methods:**

An online questionnaire was conducted among HCWs of the City of Helsinki and Helsinki University Hospital between 12.6.2020 and 5.4.2021. Altogether 1,580 HCWs participated in the study, from whom 579 shared 1,666 free-text responses. These responses were analyzed qualitatively, and the results were combined with statistical data on the participants' working conditions and backgrounds.

**Results:**

We identified problems in PPE availability and changing guidelines as factors causing the most distress in the participants. Regarding availability, running out of masks and respirators emerged as the most worrying issue, and inadequate PPE was associated with the excessive workload (OR 1.51, CI 95% 1.01–2.25). The results also highlight the importance of transparent and clear communication regarding IPC instructions and guidance, and clear IPC guidance was associated with better levels of reported recovery from work (OR 1.51, CI 95% 1.06–2.14).

**Conclusions:**

Our study highlights the importance of adequate PPE provision, transparent communication, clear guidance, and supportive supervisory work in this ongoing pandemic and potential new ones. We suggest more rigorous preparation, with crisis communication planning and emergency storage of PPE.

## Introduction

A career as a healthcare professional is commonly perceived as a calling and a profession for life (1). Past decades have brought challenges in the working conditions and work-related wellbeing of healthcare workers (HCWs), however, and the COVID-19 pandemic has exacerbated the pre-existing epidemic of burnout (2–9). Inherent in the pandemic are several risk factors related to emotional distress, such as exposure to the virus, fear of infecting friends and family, limited treatment options, longer working hours and shortages of personal protective equipment (PPE) (10–14). Some even foresee a wave of mass redundancies in the future unless major changes are made in the working environment of HCWs (15).

A state of emergency was declared in Finland in mid-March 2020, due to COVID-19 (16). Several restrictions were imposed, focused on social distancing. The District of Helsinki and Uusimaa (HUS, Helsinki University Hospital) had the heaviest disease burden in Finland, with an incidence of ~311 cases/100,000 residents during the first wave between March and June 2020 (17). The number of infections and deaths remained low in Finland during the first waves, among both the general population and HCWs (18, 19). However, given their frequent contact with COVID-19 patients and colleagues, HCWs were clearly at greater risk of infection than the general population, possibly affecting their wellbeing (20).

It was essential to protect HCWs from infection during the pandemic, key elements of which included the provision of adequate infection prevention and control (IPC) guidelines, along with PPE. It has been shown that both PPE provision and clear IPC guidelines are linked to the wellbeing of HCWs during epidemics (11, 13, 21, 22). With this study, we share more detailed insights into challenges regarding PPE use and IPC guidance during the COVID-19.

A qualitative approach allows a broader narrative and the identification of factors that might be overlooked in forced-choice questions. In our large-scale survey-based qualitative study, therefore, we analyzed 1,666 free-text responses from 579 participants and combined the results with the statistical data. The purpose was to enhance understanding of the experiences and feelings of HCWs during the COVID-19 pandemic, with specific reference to IPC practices and guidance, focusing on the quality and availability of PPE, guidelines, and management. Our aims in this comprehensive qualitative analysis are to enhance future guidance, improve the working conditions of HCWs, and ensure that institutions are ready to operate in unexpected crises.

## Materials and methods

The purpose of this descriptive phenomenological study and statistical analysis is to shed light on the lived experiences of first line HCWs during the first and second waves of the COVID-19 pandemic in Finland. We, therefore, analyzed the experiences and feelings of HCWs in primary and tertiary healthcare concerning IPC practices and guidance, focusing on the quality and availability of PPE, guidelines, and management.

### Participants

Participants were recruited *via* mass e-mailing and messages posted on the intranet of primary and tertiary healthcare organizations in the City of Helsinki (CH) and Helsinki University Hospital (HUS). Before being accepted, all volunteers were asked to read the study information and to give their informed consent, confirmed by means of strong electronic identification. The inclusion criteria were being employed by CH or HUS, working in healthcare facilities as a professional with direct patient contact, and being over 18 years old.

### Questionnaire

The survey was conducted between 12.6.2020 and 5.4.2021 via an online questionnaire among primary (CH) and tertiary (HUS) HCWs. The original questionnaire was constructed-for-purpose and comprised 50 questions in HUS and 54 in CH, from which three of the four open-ended questions were included in the qualitative analysis (Table 1). Participants were asked to describe their experiences, feelings and working conditions starting from 16.3.2020, when the first COVID-19 restrictions were announced in Finland. However, given the longer permission-granting process in the primary healthcare sector, the questionnaire was available to tertiary HCWs 6 months earlier than to primary HCWs.

**Table 1:** Free-text questions included in the analysis.

1. If there have been significant changes in how you use your personal protective equipment during the epidemic, you can open the stages here (please indicate both the protective equipment used and the time (e.g., March)]?
2. If you reported any abnormalities in access to protective equipment, at what time and at what point?
3. Is there anything else you would like to share?
4. Would you like to give more information about your welleing? In what ways have you independently sought to alleviate the symptoms caused by any increased workload, and have you experienced any of these means/activities as helpful?

### Qualitative analysis

**Figure 1:**
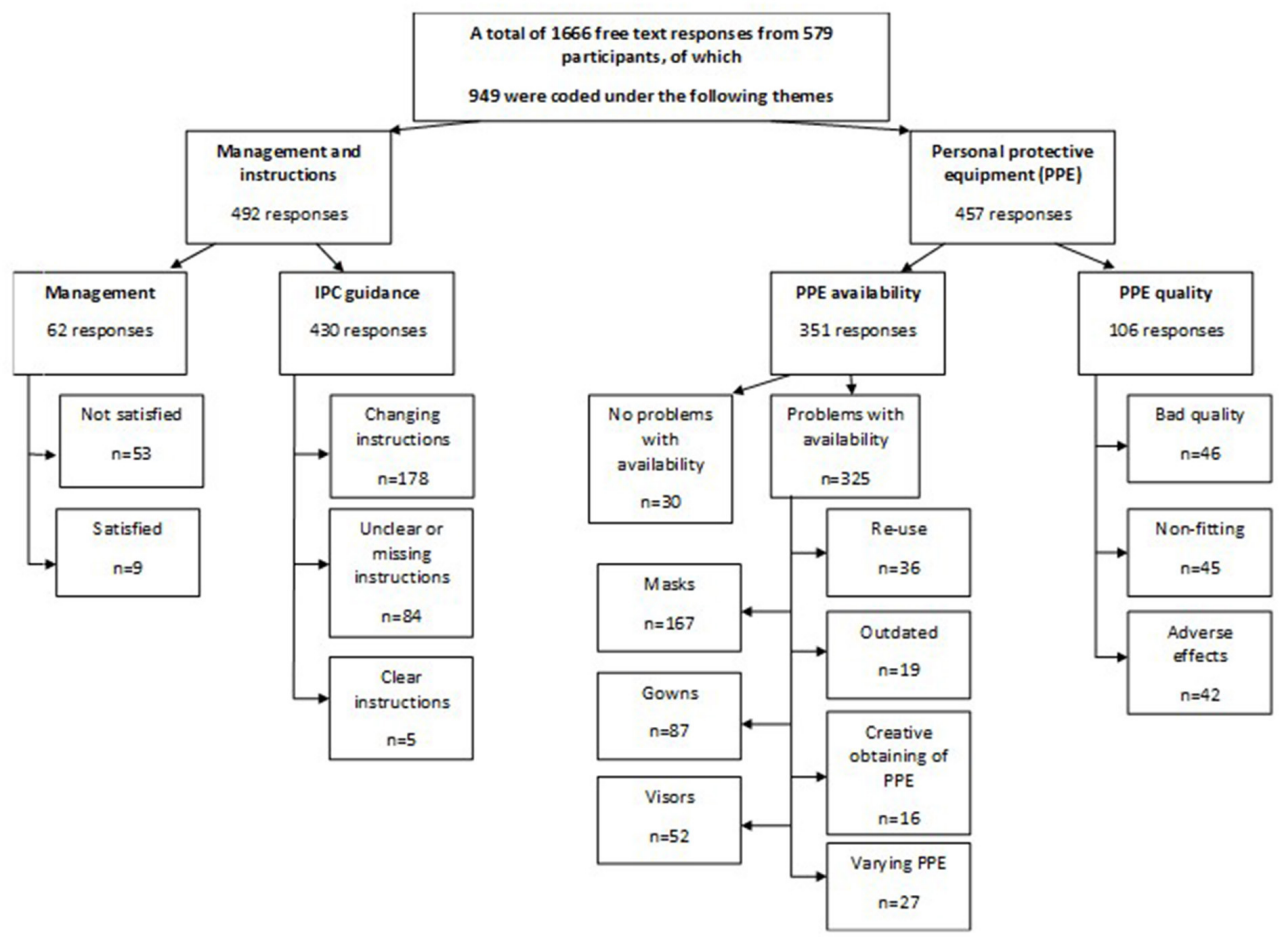
Flow chart.

The qualitative analysis was based on three open-ended questions from the original questionnaire. A modified version of Colaizzi's phenomenological method was used. In our study, the analysis was not returned to the participants for validation given its pseudonymized nature and the numbers involved. After the data collection, first, one author analyzed the data and divided it between two thematic subgroups using QSR NVivo 12 software. Second, two authors simultaneously independently scrutinized the subdivided material and, after a meeting to achieve consensus, further divided the data into smaller topics according to the themes emerging in the free-text responses (Figure 1). Third, one author further analyzed the content of the responses and integrated the results into a manuscript. When analyzing the results, a single response may fit into more than one category, hence the total number of responses in the subcategories may be greater than the number of original responses. The direct quotations in the text have been translated from Finnish into English.

Participants were not required to give free-text responses. With questions 1 and 2, the aim rather being to allow additional information to be provided regarding PPE use, as valuable insights from first-line workers could easily have been overlooked had the questionnaires comprised only forced-choice questions. Although there were no direct questions related to management, the topic emerged as a recurring theme mainly in question 3, with 62 participants sharing their insights. Thus, we felt that there was a message worth sharing and conducted a material-based analysis of these answers.

### Quantitative analysis

**Figure 2:**
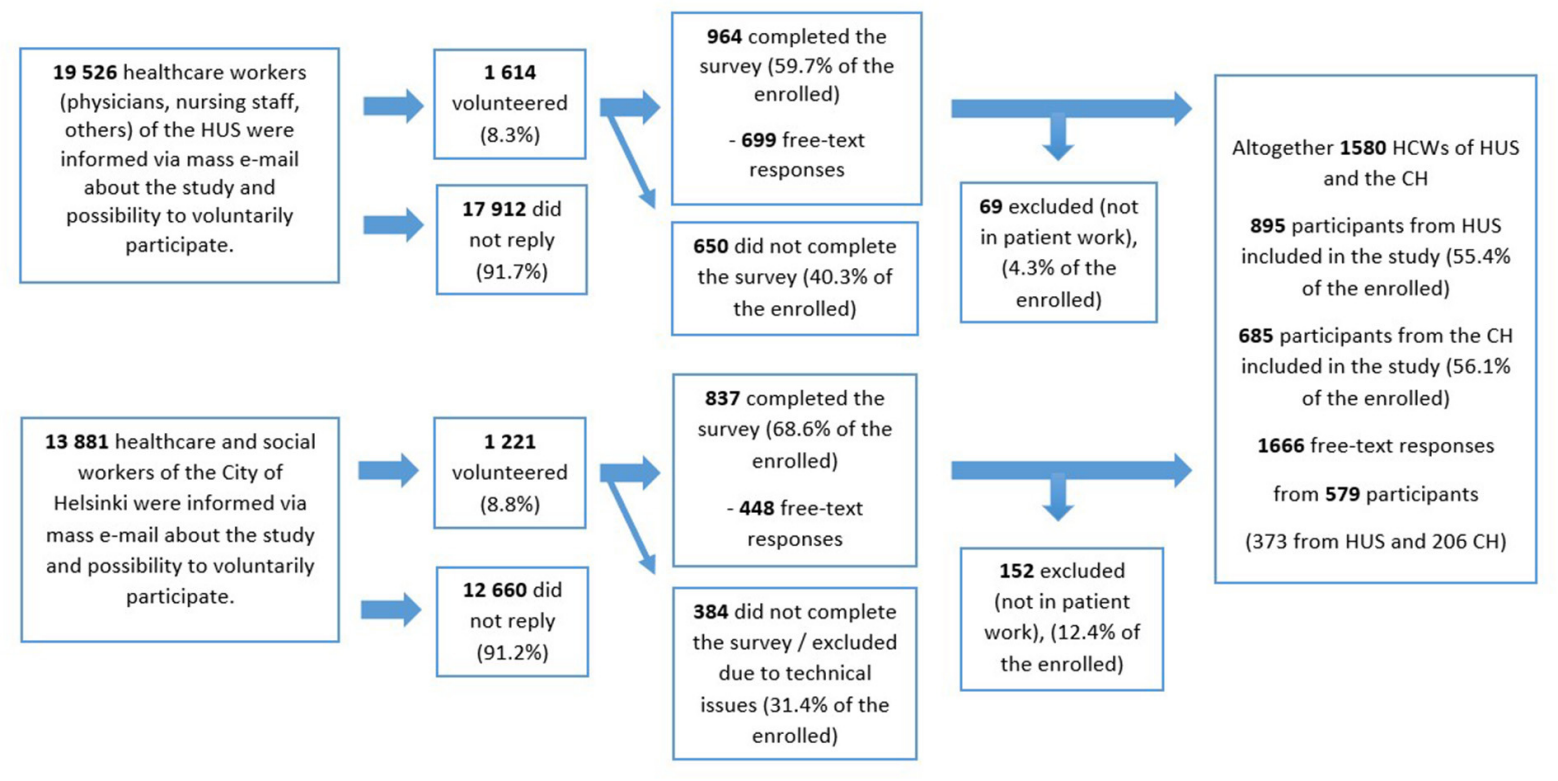
Questionnaire survey of workers at Helsinki University Hospital (HUS, tertiary healthcare) and the City of Helsinki (CH, primary healthcare).

Quantitative data were collected as part of a comprehensive study on the working environment and wellbeing of HCWs (20, 23) (Participant flow chart for the entire study, see Figure 2). The quantitative analysis covered 14 questions about the participants' background and working conditions. We used IBM SPSS for Windows 25 (IBM Corp., Armonk, NY), specifically the chi-square test, and Fisher's exact and binary logistic regression with a significance level of *p* < 0.05.

### Ethical considerations

All procedures involving human participants were in accordance with the ethical standards of the institutional research committee and the 1964 Declaration of Helsinki and its amendments, or comparable ethical standards. The Ethical Committee of HUS (HUS/1450/2020) and the CH (HEL 2020-007596 T 13 02 01) approved the study protocol. All the analyzed responses were anonymized.

## Results

In total, 579 participants shared 1,666 free-text responses to the three questions we had chosen to analyze. Of these, 949 were related to PPE, IPC guidance and management and therefore analyzed further. The participants represented a wide range of HCWs with 88 (15.2%) doctors and dentists, 346 (59.8%) nurses or nursing staff members and 105 (18.1%) other workers such as physiotherapists, speech therapists, psychologists, and occupational therapists. Specific occupational information was missing from 40 (6.9%) participants. The majority of participants were women, 92.4% (532/579), and were employed in tertiary healthcare (HUS) 64.4% (373/579) (Table 2). An increased workload was reported by 60.1% (348/579). However, increased working hours were not common, reported by only 23.8% (138/579). Recovery from work was seemingly impaired, with only 36% (211/579) of participants reporting normal recovery. The content analysis revealed four main topics in the HCWs' shared experiences: (i) PPE availability, (ii) PPE quality, (iii) IPC guidance, and (iv) management.

**Table 2:** Characteristics of the participants.

***n* = 579**	***n* (%)**
**Gender**, ***n*** **=** **577**	
Women	535 (92.4)
Men	43 (7.4)
**Occupation**, ***n*** **=** **539**	
Doctors/dentists	88 (15.2)
Nursing staff	346 (59.8)
Others	105 (19.5)
**Age**, ***n*** **=** **577**	
18–29	87 (15.0)
30–39	164 (28.3)
40–49	150 (25.9)
50–59	139 (24.0)
60–69	37 (6.4)
**Comorbidities**, ***n*** **=** **575**	
Severe heart disease	2 (0.3)
Lung disease, not clinically stabilized	16 (2.8)
Diabetes involving organ injury	3 (0.5)
Disease that weakens the immune system	6 (1.0)
Immunosuppressive medication	14 (2.4)
**Smoking**, ***n*** **=** **579**	62 (10.7)
**Pregnant**, ***n*** **=** **578**	14 (2.4)
**Living conditions**, ***n*** **=** **578**	
Living alone	113 (19.5)
With one other person	222 (38.3)
With two other people	81 (14.0)
With three or more other people	162 (28.0)
**Regular medication**, ***n*** **=** **579**	279 (48.2)
**Employer**, ***n*** **=** **579**	
HUH	373 (64.4)
CH	206 (35.6)
**Has treated confirmed COVID-19 patients**, ***n*** **=** **578**	258 (44.6)

### PPE availability and quality

Altogether, 48.2% (457/949) of the responses concerned PPE availability (37.0%; 351/949) and quality (11.2%; 106/949). Masks attracted the most comments, referring to both availability and quality. Up to 74.5% (79/106) of the comments on PPE quality and 45.6% (160/351) on availability concerned masks and respirators: the corresponding figures for coats and visors were 31.1% (33/106) and 13.2% (14/106) for quality; 24.7% (87/351) and 14.8% (52/351) for availability.

Only 5.7% (6/106) of the responses concerning PPE quality, and 1.4% (5/351) of those concerning availability, were about head coverings. Responses on availability and quality were further divided into subcategories by emerging themes, as described below.

In response to the forced-choice questions, 7.1% (41/579) of the participants reported re-using PPE, and 4.8% (28/579) working without it. Up to 44.6% (258/579) reported working with inadequate PPE, which was associated with higher levels of workload (OR 1.51, 95% CI 1.01–2.25) and excess working hours (OR 2.01, 95% CI 1.24–3.24).

#### PPE availability

“*I have received PPE in accordance with the instructions, because according to the instructions, PPE was systematically lightened during the spring. For example, whereas FFP2 / FFP3 respirators were initially used in the ward (as in MERS isolation, for example), it was no longer necessary to use it at the end of March according to HUS instructions (except with aerosols). If we had been allowed to use it (as we would have liked), there would certainly have been a shortage of them.”*

PPE shortages and concerns about running out caused distress among the participants. Many HCWs felt uncertain about its availability, especially during the first months of the pandemic. Regarding PPE availability, the HCWs expressed feelings of fear, vulnerability, confusion, annoyance, discomfort, and not being heard. Most responses (92.6%; 325/351) described problems with availability. Re-using disposable products (10.3%; 36/351), outdated products (5.4%; 19/351), varying products (7.7%; 27/351) and creative procurement (4.6%; 16/351) were re-emerging themes in responses related to availability. Visors appeared to be the most commonly re-used items (41.7%; 15/36), and several comments referred to broken visors, and impaired vision due to their re-use. Running out of FFP respirators and surgical masks and replacing them with masks with lower protection properties were common worries. Nevertheless, working entirely without PPE against recommendations did not appear to be prevalent. Some participants mentioned that FFP respirators were sometimes available for doctors but not for nurses, and that items such as respirators were locked in a cabinet and not available to HCWs engaged in laboratory and imaging work. Shortages of masks, gowns, visors, and caps were most referred to, but insufficient hand disinfectant, gloves, shoe covers, and other types of eye protection were also mentioned.

Restrictions on PPE usage (e.g., up to four masks per day), limitations on breaks, not being allowed to change PPE between patients and long periods using the same PPE were also recurring themes (8.3%; 29/351 responses). However, participants also showed creativity in coping with the shortages: some of them assembled PPE from plastic screens and combined different types of equipment to have adequate protection. Several also described purchasing PPE from stores or pharmacies, obtaining it from different wards and units, and even organizing trafficking between units.

#### PPE quality

“*The protective gowns broke when put on. It did not protect us from vomit or other fluids as secretions came through to the skin.”*

Of the 106 responses on PPE quality, 43.4% (46/106) concerned poor quality, 42.5% (45/106) poor fit and 39.6% (42/106) adverse effects. Having to use it constantly affected the HCWs' work-related wellbeing, and they associated a wide range of adverse effects with its use. The most common of these were skin problems (40.5%; 17/42), allergic reactions (28.6%; 12/42), foul odor (26.2%; 11/42) and breathing problems (26.2%; 11/42). Excess sweating, impaired vision, headache, sore throat, loss of voice, dry mouth, ulcers on the face, blocked nose and swollen eyes were also mentioned. The respondents also referred to breaking and fragile materials, foul-smelling masks and masks smelling of mold, hard or thin, uncomfortable materials, along with deteriorating quality as the epidemic went on. Problems with fit were most apparent in masks and respirators (47.6%; 20/45) and gowns (42.2%; 19/45). Regarding PPE quality the HCWs expressed feelings of suspicion, annoyance and fear.

### Infection prevention and control guidance and management

Our content analysis revealed that up to 492/949 (51.8%) of the free-text responses concerned IPC management and guidance (45.3%; 430/949 on guidance and 6.5%; 62/949 on management). In response to the forced-choice questions, 94% (544/579) of the participants reported having received PPE training, and 90.8% (526/579) felt they knew how to don and doff properly. Guided training was offered to 36.1% (209/579), and 38% (220/579) found the IPC guidance clear: Clear guidance was associated with better reported levels of recovery from work (OR 1.51, 95% CI 1.06–2.14).

#### Infection prevention and control guidance

“*Absolutely the most burdensome aspect of the whole epidemic was the ever-changing guidelines, many of which conveyed that even the authors did not know what to do and therefore ended up with some kind of panic solution that had no head or tail.”*

Several participants mentioned frequently changing guidelines as the most burdensome factor of the pandemic, and up to 41.4% (178/430) of the responses referred to frequently changing instructions. A notable cause of the distress was the non-transparency of communication, which aroused suspicions of dishonesty or lying behind the guidance (6.7%; 29/430). Regarding IPC guidance, the HCWs described feeling neglected, and feelings of stress, fear, confusion, uncertainty, not being protected, and not being heard. There were also references to unclear or missing guidelines (19.5%; 84/430), and clear guidelines (1.2%; 5/430). However, although reporting changing and unclear instructions at the beginning of the pandemic, many participants felt that the situation improved during its course.

“*We had the impression at first that protective equipment was not needed, but when it became more available, it was recommended to use it despite no real change in the situation of the pandemic.”*

Unequal treatment of employees depending on the job or unit was highlighted in 3.0% (13/430) of the responses. For example, the guidelines differed in different units, such as the laboratory, imaging, and home hospital. In addition, according to some participants, initially there were no instructions related to the specific circumstances of their unit, namely operating rooms, and obstetrics, for example. Guidelines were also apparently non-uniform: some participants working in several wards reported that they differed.

“*The guidelines changed so often that I couldn't stay on the page about the right way. Aseptic conscience has had to be suppressed because we must act against the instructions that we have learned are correct.”*

Working against their own work ethic arose as a significant issue in the participants' responses: directions and guidance were not in accordance with what they had learned, and questioning and disobedience were not tolerated. They also highlighted the following issues: (i) feeling unsafe as HCWs were directly prohibited from wearing PPE at the beginning of the pandemic, despite their wishes; (ii) instructions about social distancing caused frustration because they could not be followed given the small and crowded working areas; (iii) instructions to don and doff in the rooms of COVID-19 patients; (iv) suspicions that HCWs had been infected due to inadequate IPC guidance.

#### Management

“*The situation has not been made easier by the employer's complete lack of support and understanding in a difficult situation.”*

Dissatisfaction with management and supervisors was a notable theme in the survey: 85% (53/62) of participants sharing insights on leadership during COVID-19 were dissatisfied with the performance of their employer and supervisors.

“*There has been a feeling that no one wants to protect us.”*

In this stressful novel situation, lack of support from supervisors (16.1%; 10/62) emerged as the strongest management-related stress factor. Other re-emerging issues included a lack of compensation on the institutional level, the cancellation of holidays, feeling neglected and the feeling of not being heard. In addition, the HCWs described feelings of confusion, frustration, fear, stress, shock, dissatisfaction, anxiety, and annoyance. Several HCWs suspected that lies and disinformation were behind the changing IPC guidelines and expressed frustration with the non-transparent decision-making. Questioning and accusations from supervisors, their lack of appreciation, belittling and negligence were clearly present in the participants' descriptions of their experiences. On the other hand, many were grateful for the support they had received from colleagues, and 14.5% (9/62) of the participants spontaneously shared positive experiences with management and supervisors during the pandemic.

## Discussion

“*We hope lessons will be learned, and that instructions will be given in advance in situations like this. You should not need to fight for protective equipment to do your job, you should use your energy for work. This created a really nasty atmosphere in our workplace. Some of the staff thought we didn't matter.”*

Our findings give valuable insights into the experiences and feelings of HCWs during the first waves of the COVID-19 pandemic. We identified problems in PPE availability and changing guidelines as factors causing the most distress among the participants. In terms of availability, running out of masks and respirators emerged as the most worrying issue, and inadequate PPE was associated with the excessive workload. The results also highlight the importance of transparent and clear communication regarding IPC instructions and guidance. Dissatisfaction with management related to a lack of support from supervisors, and clear IPC guidance was associated with better levels of recovery from work.

### PPE availability and quality

“*I wish HUS had justified the constant changes in PPE practices somehow and not always just changed the guidelines. We nurses were always forced to work according to HUS's guidelines, and they were constantly changing for the worse, for no good reason. If we had talked openly at work about the lack of protective equipment, perhaps the nurses would have been even more understanding. It was as if our health didn't matter.”*

Problems in PPE availability emerged as a major distress factor among the participants. Other recurrent themes included re-use, deterioration in quality, non-fitting PPE, adverse effects, and uneven distribution. Furthermore, working with inadequate PPE was associated with a reportedly more burdensome workload and excess working hours.

According to WHO estimates, by the end of May 2021, the COVID-19 pandemic had claimed the lives of up to 180 000 HCWs (23). Given that infections among HCWs are associated with inadequate PPE and close contact with COVID-19 patients (20, 24, 25), it is understandable that PPE shortages are associated with higher levels of emotional distress (11, 13, 26), not to mention the fear of infecting loved ones. Our findings confirm the association between PPE shortages and perceived distress among HCWs (27).

The rapid increase in PPE demand and the limited stocks challenged healthcare systems globally at the beginning of the pandemic (28). As a result of the limited availability, the quality of protection deteriorated because the equipment was purchased from wherever it was available (29–31). Many participants suspected that IPC guidance was dependent on PPE availability: when something was unavailable, suddenly it was no longer necessary. If the guidelines really were based on availability rather than the latest scientific research and best understanding, then the principles of evidence-based medicine were not followed. As a result, the wellbeing of HCWs was put at risk. Adverse effects were also frequently mentioned in connection with PPE. Our participants described several, and as previous studies have shown, they are not uncommon (32, 33). PPE is essential to reduce the risk of nosocomial infection among HCWs (34), thus products of adequate quality, comfortable fit and minimal adverse effects should always be available.

### IPC guidance and management

“*IPC information should be centralized. Now guidance has come from various sources. Organizational structures should be followed even during an epidemic, and information should flow normally.”*

We identified frequently changing guidelines as the factor causing participants the most distress related to IPC guidance and management, their responses clearly indicating distrust and dissatisfaction. Over 60% of them mentioned problems with instructions or guidance. Furthermore, we found that clear IPC guidance was associated with better recovery from work.

Earlier studies on COVID-19 and previous epidemics have revealed the importance of clear IPC instructions, guidelines and organizational support (11, 22, 35, 36). It has been shown that unclear instructions and frequently changing guidelines cause confusion and reduce levels of trust in their reliability (11, 22, 35), whereas transparent communication and the provision of adequate PPE information have been associated with higher safety perception (21, 28).

The beginning of the pandemic was characterized by a scarcity of information on COVID-19, including the mode of transmission and adequate means of protection. The guidelines were based on information about previous pathogens and were updated as knowledge of the virus increased. It is not surprising, therefore, that changing instructions were identified as a recurrent theme in the survey. The desire for clarity and uniformity in the instructions was reflected in the responses. However, it is not realistic to expect fully consistent guidelines given the different infection risks in different departments and units.

Several studies have highlighted the importance of organizational support and transparent communication for the psychological wellbeing of HCWs who put themselves at risk (36–38). They understand that masks and FFP2 and FFP3 respirators are necessary when they carry out aerosol-generating procedures or work in a cohort unit, for example, and thus accept PPE availability prioritization for this unit. However, when communication lacks transparency, and workers only see the discrepancy in PPE availability, they tend to feel they are being treated unequally and even neglected.

The COVID-19 pandemic has put us all under unseen pressure, especially healthcare systems, and decisions had to be made based on best estimates. However, hospitals and other organizations did have prepared guidelines in the event of unknown infectious diseases. Because of previous experience with SARS and following the precautionary principle, these guidelines did consider the airborne transmission. However, problems with PPE provision prevented some of the precautionary guidelines from being implemented. Our study highlights the need for the rationale behind protection guidelines to be scientific and transparent. Communication needs to be clear and honest, because only then can there be trust on both sides.

### Strengths/weaknesses

Our study has several strengths, one being the large sample size, which makes it comprehensive, especially compared with other qualitative studies. In addition, our study population is comprehensive in that the participants represent broadly different occupations and clinics in primary and tertiary healthcare. The majority of qualitative studies on the working conditions of HCWs are limited in sample size and to one hospital or unit. Furthermore, our survey was conducted during the first two waves of the pandemic, which allowed us to include fresh, up-to-date insights. Another strength is the added statistical data, which improves the quality and trustworthiness of the results.

Given the pseudonymous nature of our survey, we believe we have obtained honest answers, with no fear of adverse consequences as a result of criticizing management and its practices. On the other hand, free-text responses provide limited information. Moreover, voluntary participation may cause bias in the results, which must not be forgotten in the analyses. Participants who had experienced challenges in working conditions such as PPE shortages and poor management may have been more likely to share their insights. Because the questionnaires were available at different time points for HUS and CH participants, the responses are not totally comparable, as the course of the pandemic may have affected the participants' insights. Therefore, the responses from HUS HCWs may reflect experiences during the first wave of the pandemic, whereas those from CH reflect the first year. Although comprehensive, our study population still represents HCWs from a limited area and similar healthcare systems. Therefore, our results are not generalizable.

## Conclusion

Our study highlights the importance of adequate PPE provision, transparent communication, clear guidance, and supportive supervisory work in this ongoing pandemic as well as in potential new ones. We suggest more rigorous preparation, with crisis communication planning and emergency storage of PPE. Challenges in PPE provision were a global concern during the first waves of COVID-19. However, dissatisfaction with IPC guidance and management could have been prevented on an institutional level. HCWs' feelings of stress and of not being supported nor protected are to be taken seriously.

## Data availability statement

The datasets presented in this article are not readily available because dataset only pseudonymized. Requests to access the datasets should be directed to enni.sanmark@hus.fi.

## Ethics statement

The studies involving human participants were reviewed and approved by Helsinki University Hospital Ethics Committee. The patients/participants provided their written informed consent to participate in this study.

## Author contributions

IA, PN, ES, and AG contributed to the conception and design of the study. LO, SO, LL, MP, SP, AK, PP, ES, and AG contributed to the design of the survey and data collection. IA and PN performed the qualitative analysis. IA performed the statistical analysis and wrote the first draft of the manuscript. All authors contributed to manuscript revision, read, and approved the submitted version.

## Funding

IA has received funding from the Tampere Tuberculosis Foundation, the Medical Society of Finland, the Väinö and Laina Kivi Foundation, and the Helsinki University Hospital Research Fund and also supported by the City of Helsinki. LL has received funding from Finnish Dental Society Apollonia and the Finnish Women Dentists' Association.

## Conflict of interest

The authors declare that the research was conducted in the absence of any commercial or financial relationships that could be construed as a potential conflict of interest.

## Publisher's note

All claims expressed in this article are solely those of the authors and do not necessarily represent those of their affiliated organizations, or those of the publisher, the editors and the reviewers. Any product that may be evaluated in this article, or claim that may be made by its manufacturer, is not guaranteed or endorsed by the publisher.
